# Clinical phenotype of neonatal lupus erythematosus relates to autoantibody level and gender

**DOI:** 10.1186/1546-0096-9-S1-O14

**Published:** 2011-09-14

**Authors:** S Venkatesan, NG Lawrence, C Carbone, E Jaeggi, ED Silverman, S Kamphuis

**Affiliations:** 1 Department of Rheumatology, Sophia’s Children Hospital, Erasmus University MC Rotterdam, The Netherlands; 2 Department of Rheumatology, The Hospital for Sick Children, Toronto, Canada; 3 Department of Cardiology, The Hospital for Sick Children, Toronto, Canada

## Background

Neonatal Lupus Erythematodus (NLE) is a rare disease occurring in offspring from mothers with anti-Ro with or without anti-La antibodies. Much is known about the individual manifestations (congenital heart block (CHB), cutaneous rash, hematologic and hepatic laboratory abnormalities and macrocephaly) but it is unclear how these and other autoantibodies (anti-dsDNA, anti-RNP and anti-Sm antibodies) influence disease phenotype.

## Aim

To analyze the frequency and characteristics of clinical phenotypes seen in NLE and relate these phenotypes to the quality and quantity of autoantibodies.

## Methods

A cohort of 261 infants whose mothers had anti-Ro antibodies was followed prospectively at The Hospital for Sick Children. All infants underwent the first full clinical evaluation and laboratory testing between 4-12 weeks after birth.

## Results

A small majority of infants (150/261, 57%) had one or more NLE manifestations consisting of 56 (37%) with a cutaneous rash, 44 (29%) had liver function abnormalities, 42 (28%) had CHB, 41 (27%) had neutropenia (<1.0x10^9^/l), 21 (14%) had macrocephaly and 9 (6%) had thrombocytopenia. CHB was an isolated finding in more than half of the cases (26/42), as was neutropenia (23/41); the other NLE manifestations most often presented in combination. Where the cohort of NLE patients had slightly more females (59% versus 41% males), a larger majority of patients with CHB were females (74%), but macrocephaly was seen more in males (62%). The chance of developing NLE was associated with increasing levels of anti-Ro antibodies. Macrocephaly was only seen in infants that still had a positive anti-Ro titer when sampled after birth. Increasing levels of anti-La antibodies were associated primarily with cutaneous rash. Anti-dsDNA, anti-RNP and anti-Sm antibodies were present in less than 5% of infants and could not be related to clinical phenotype.

## Conclusion

In this large cohort of prospectively followed infants from mothers with anti-Ro antibodies, a majority developed NLE. The clinical phenotype of NLE appeared to be related to gender and to the quantity of anti-Ro and -La antibodies.

